# Randomised clinical trial evaluating best-corrected visual acuity and central macular thickness after 532-nm subthreshold laser grid photocoagulation treatment in diabetic macular oedema

**DOI:** 10.1038/eye.2015.1

**Published:** 2015-02-20

**Authors:** W Pei-pei, H Shi-zhou, T Zhen, L Lin, L Ying, O Jiexiong, Z Wen-bo, J Chen-jin

**Affiliations:** 1 State Key Laboratory of Ophthalmology, Zhongshan Ophthalmic Centre, Sun Yat-sen University, Guangzhou, China

## Abstract

**Purpose:**

To compare best-corrected visual acuity (BCVA) and central macular thickness (CMT) after 532-nm subthreshold laser grid photocoagulation and threshold laser grid photocoagulation for the treatment of diabetic macular oedema (DME).

**Patients and methods:**

Twenty-three patients (46 eyes) with binocular DME were enroled in this study. The two eyes of each patient were divided into a subthreshold photocoagulation group and a threshold photocoagulation group. The eyes of the subthreshold group underwent 532-nm patter scan laser system (PASCAL) 50% end point subthreshold laser grid photocoagulation therapy, whereas the threshold photocoagulation group underwent short-pulse grid photocoagulation with a 532-nm PASCAL system. BCVA and CMT were assessed in all patients before treatment, 7 days after treatment, and 1, 3, and 6 months after treatment.

**Results:**

After grid photocoagulation, the mean BCVA improved in both the subthreshold group, and the threshold group, and the two groups did not differ statistically significantly from each other. Similarly, the macular oedema diminished in both groups after treatment, and the two groups did not differ statistically significantly from each other with regard to CMT.

**Conclusion:**

Both 532-nm subthreshold laser grid photocoagulation and threshold laser grid photocoagulation can improve the visual acuity and reduce CMT in DME patients.

## Introduction

Diabetes affects over 220 million people worldwide and diabetic retinopathy (DR) is the most common cause of vision loss in working-aged individuals in developed countries.^1, 2, 3^ In this context, moderate vision loss is mainly due to diabetic macular oedema (DME), which arises from leakage of plasma into the central retina. It has been reported that in a cohort of patients who had been diabetic for 20 or more years, 29% suffered from DME.^4^ The progressive plasma leakage leads to thickening of the macula structure and distortion of neurons. Visual acuity is reduced irreversibly over time, as the neurons involved die.

Though the pathogenesis of DR remains uncertain, it is generally accepted that hyperglycaemia is the primary cause, due to the association between poor glycaemic control and retinopathy progression.^5^ The damage to retinal blood vessels arising from hyperglycaemia leads to microaneurysms and capillary closure. The leakage of microaneurysms contributes substantially to the development of DME.^6^ A pilot study has demonstrated that hypoxia is implicated in the pathogenesis of DME.^7^ This finding may partly explain the occurrence of diffuse DME in the absence of leakage of microaneurysms. Hypoxia stimulates the expression of vascular endothelial growth factor (VEGF), which enhances vascular permeability, potentially leading to leakage of retinal vessels.

The Early Treatment Diabetic Retinopathy Study (ETDRS)^8^ demonstrated that laser photocoagulation for clinically significant macular oedema helped to reduce the incidence of visual loss by ∼50% at the 3-year follow-up. The most common mode of laser photocoagulation treatment for DME is macular laser grid photocoagulation.^8, 9^ However, traditional photocoagulation with grey or grey-white laser spots inevitably scars the retina, destroys photoreceptors, and is associated with vision scotoma.^10, 11, 12^ The parameters of laser photocoagulation have evolved in recent clinical studies, which have incorporated shorter pulse durations and lower laser energy settings, leading to paler laser spots.^13^ Clinical trials have demonstrated that lower-energy laser photocoagulation can reduce side effects and risks, while effectively alleviating DME.^14, 15, 16^

In order to stimulate a retinal healing response and improve the accuracy and safety of laser photocoagulation, we conducted a randomised clinical trial to compare the 532-nm threshold and subthreshold laser grid photocoagulation for the treatment of DME, with best-corrected visual acuity (BCVA), and central macular thickness (CMT) as observation indices.

## Materials and methods

This study was a prospective, 1 : 1 matched, randomised clinical trial that involved initial laser therapy for treatment-naive DME patients, with 6 months follow-up, undertaken at the Zhongshan Ophthalmic Centre of Sun Yat-sen University, between September 2011 and June 2013. Inclusion criteria are as follows: (1) Aged >18 years; (2) Patients with diabetes mellitus type 2 who meet the WHO or ADA criteria for diabetes; (3) ETDRS visual acuity ≥19 letters (Snellen equivalent of 20/400 or better); (4) Newly diagnosed SNPDR; (5) Mean CRT of more than 300 *μ*m as measured by optical coherence tomography (OCT) scans; (6) Adequate pupil dilatation and clear media to perform laser photocoagulation and OCT. Exclusion criteria include (1) Planned PRP within 6 months; (2) Previous intraocular surgical or laser treatment to the study eye within 6 months; (3) Previous retinal treatment: laser, drug or surgery; (4) Previous laser photocoagulation or macular laser treatment to the study eye; (5) Any previous ocular condition that may be associated with a risk of macular oedema; (6) Uncontrolled hypertension, renal failure, and mental illness; (7) Planned insulin therapy within 6 months because of poor glycaemic control; (8) Planned intraocular surgery within 6 months. The study protocol was registered at Clinical Trial.gov (NCT01759121) and Chinese Clinical Trial Registry (ChiCTR-TRC-12002735). It was approved by the relevant research ethics committee (2012KYNL056), and written informed consent was obtained from all participants. We certify that all applicable institutional and governmental regulations concerning the ethical use of human volunteers were followed during this research.

All participants received detailed ophthalmic examinations at baseline and at follow-up visits at 7 days, and 1, 3, and 6 months. The age of each subject was recorded, as were the following clinical parameters: duration of diabetes mellitus, average fasting blood glucose 2 weeks before laser photocoagulation therapy, and recent glycosylated haemoglobin level within 3 months. The clinical data collected included BCVA as measured by the ETDRS vision chart at 2 m, type and severity of DME, and the total number, energy, time of exposure, and spot diameter of subthreshold or threshold laser photocoagulation burns applied to the treated eye.

Patients were identified as having diffused DME with central fovea involved at the stage of severe non-proliferative DR by fundus fluorescein angiography (FFA). At the baseline and follow-up time points of the trial, patients underwent OCT to assess average CMT within a scope of 1 mm in diameter.

The study was internally controlled because both the treatments were applied in the same patient—one to each eye. All patients with a mean CMT of >300 *μ*m underwent grid pattern laser therapy, via a 10-ms-pulse PASCAL (Topcon Medical Laser Systems, Santa Clara, CA, USA) photocoagulation with MAC A+MAC B and single-spot type performed by an experienced ophthalmologist. Threshold grid pattern laser photocoagulation was titrated to, and designated by, a light grey burn (between grade 1 and 2) in accordance with ETDRS guidelines, then three rings of laser burns at the same intensity were pattern scanned at a distance of macular central fovea over 500 μm. Subthreshold photocoagulation was titrated to a light grey burn (between grade 1 and 2), but other pattern scanned laser burns were applied at 50% energy of the titrated spot, which was invisible to the naked eye. All treatments were performed by PASCAL, except titrated spots under topical 0.5% proxymetacaine hydrochloride, for which 10-ms and 60-*μ*m macular grid PASCAL multispot arrays were used. Initial energy was 75 MW, and increases of 25 MW were utilised as needed. Burns were applied one burn width apart, more than 500 *μ*m temporal to the fovea.

Primary outcome measures included (1) post-laser visual acuity as determined by the ETDRS vision chart and (2) average CMT as determined by OCT. Secondary outcome measures included DME area, laser burn dosimetry, and intensity of the laser spot reaction.

All patients underwent macular spectral domain OCT retinal thickness measurements (Spectralis, Heidelberg Engineering, Heidelberg, Germany). The OCT image was based on the average of 100 scans, derived using an automatic averaging function to reduce speckle noise and a real-time eye tracking system. Mean CMT was measured as the average macular thickness within a scope of 1 mm in diameter, centred around the fovea.

We performed statistical analysis using SPSS16.0 (SPSS Inc., Chicago, IL, USA). Variables are presented as mean±SD. A mixed linear model was used due to missing values in follow-up periods. Changes in CMT and BCVA between different time points and baseline within each treatment group were compared via the Wilcoxon test. The Mann–Whitney *U*-test was used to evaluate differences in the changes in CMT and BCVA from baseline between the two treatment groups. The null hypothesis was rejected for *P* values <0.05.

## Results

A total of 46 eyes of 23 patients were initially recruited into the clinical trial, and the patients included 11 males (47.83%) and 12 females (52.17%) with an average age of 61.61 years (range 47–73). Thirty-one eyes (67.39%) revealed diffuse retina oedema and others (32.61%) revealed cystoid macular oedema through FFA, and *n* macular ischaemia was detected. Twenty-two eyes (47.83%) were with macular hard exudates. The follow-up study included 42 eyes of 21 patients, as two patients (four eyes) from the initial cohort were lost to follow-up (one died 2 months before completion of the clinical trial and the other relocated to another city). Fourteen eyes with residual oedema (eight eyes of subthreshold group and six eyes of threshold group) accepted repeat treatments. Five eyes (two eyes of subthreshold group and three eyes of threshold group) with more severe macular oedema after photocoagulation accepted intravitreal anti-VEGF therapy. The primary and secondary outcome measures were analysed in the two groups according to baseline BCVA and CMT. The patient characters are presented in Table 1.

### Visual acuity

**Figure 1:**
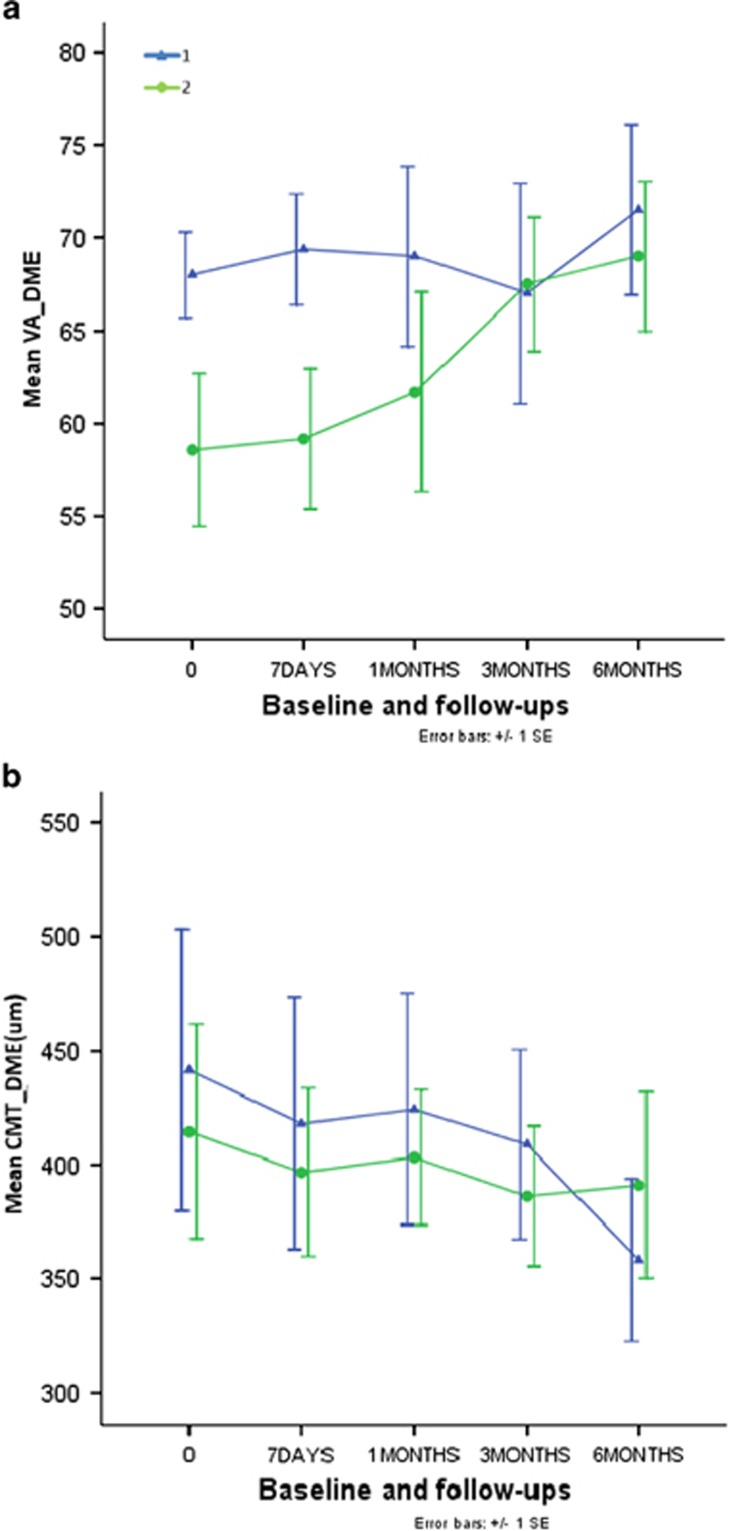
(a) Mean BCVA at baseline and follow-ups. 1, subthreshold laser photocoagulation group; 2, threshold laser photocoagulation group. *x* axis: follow-up time points; *y* axis: mean BCVA of ETDRS visual chart. (b) Mean CMT at baseline and follow-ups. 1, subthreshold laser photocoagulation group; 2, threshold laser photocoagulation group. *x* axis: follow-up time points; *y* axis: BCVA of ETDRS visual chart.

The baseline BCVA of the two groups combined was 55 (15–85) and at the final follow-up (6 months) it had improved to 58.5 (18–84). Grid pattern laser therapy yielded improved BCVA at 6 months follow-up in all patients in the study. In group 1 (subthreshold photocoagulation), the mean baseline BCVA was 60 (range 18–83) and in group 2 (threshold photocoagulation) it was 51 (range 15–85). In group 1, 2 (9.52%) patients' BCVA declined more than five ETDRS test objects, 10 cases (47.62%) improved more than five ETDRS test objects, and others (42.86%) maintained stability (0±4 ETDRS test objects). There was an early decrease at the 7-day and 1-month follow-ups. However, there was an improvement in BCVA at the 6-month follow-up (4.03±9.02, *P*=0.035). In group 2, there were 4 (19.05%) cases having BCVA declined more than five ETDRS test objects, 11 cases (53.28%) improved more than five ETDRS test objects, and others (28.57%) maintained stability. BCVA gradually increased initially, peaking at 3.50±6.38 at the 3-month follow-up. At the final follow-up, BCVA had decreased slightly and it did not differ significantly from baseline (2.17±7.14, *P*=0.65; Table 1, Figure 1a). There was no significant difference between the subthreshold and threshold photocoagulation therapies on BCVA outcomes (Tables 2 and 3, Figure 1a).

### OCT examination of DME

All eyes in both groups revealed diffuse DME at baseline, with a mean CMT in both groups combined of 432.63 *μ*m (SD 139.77 *μ*m). The CMT of the subthreshold group (group 1) exhibited a downward trend after therapy. Compared with baseline, DME was significantly reduced at the 3-month (by 49.67±69.71, *P*=0.001) and 6-month (by 62.30±102.52, *P*=0.005) follow-ups. With regard to the threshold group (group 2), at the 7-day follow-up, the severity of DME had reduced (by 25.85±43.13, *P*=0.055), and it had risen slightly by the 1-month follow-up. After that, CMT reduced gradually. Compared with baseline, the reduction of 23.63±80.99 evident at the final follow-up was statistically significant (*P*=0.04). Both the subthreshold and threshold grid pattern laser therapy relieved DME, and there were no statistically significant differences between the two at any time point after baseline (Tables 2 and 3, Figure 1b).

### Parameters of photocoagulation

Parameters of photocoagulation were set as 60 *μ*m spot diameter and 10 ms exposure time. Number of burns (140, 57–247; 144, 90–279) and total area treated (1.36±1.03, 1.53±0.76 mm^2^) between two groups have no difference. Energy fluence of subthreshold group was 32.40±20.57 J/cm^2^, which is lower than threshold group 54.17±20.57 J/cm^2^ (*P*=0.021).

**Figure 2:**
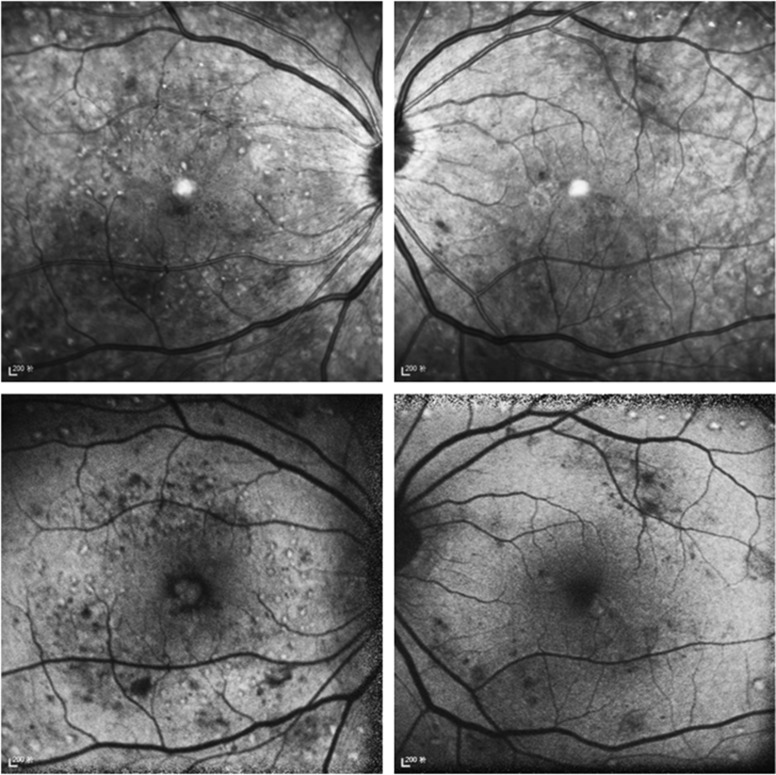
Infrared imaging (above) and autofluorescence (below) of baseline and 6 months after grid pattern laser photocoagulation. OD: threshold photocoagulation; OS: subthreshold photocoagulation.

Six months after photocoagulation, laser spots of subthreshold group are invisible to the naked eyes, as well as at infrared imaging and autofluorescence of spectral domain OCT, compared with visible laser spots at retina in threshold group (Figure 2).

## Discussion

Macular grid photocoagulation for diffuse leakage consisted of annular, 50–200 μm diameter, equally spaced laser spots greater than one half of the width of the grid pattern.^8, 9^ On the basis of several multicentre clinical trials, the understanding of laser energy for DME gradually changed from traditional full-thickness retinal photocoagulation to lower-energy photocoagulation. The aim of this clinical trial was to suppress DME, but at the same time reduce the risks of complications to the greatest extent possible. PASCAL, by which we carried out retinal photocoagulation, is a type of 532 nm, short-exposure time and exposure time-preset mode laser excitation system. The general macular settings include an exposure time of 10–20 ms; thus, the energy density and thermal diffusion are significantly reduced compared with traditional photocoagulation. Retinal photocoagulation with shortened exposure times proved not only effective for treating DME, but also for relieving patients' pain.^17^ The retinal photocoagulation induced by 10–30 ms exposure time can stimulate retinal inner healing responses, and is associated with less destructive effects than traditional methods.^18, 19^ This suggests that PASCAL can improve the accuracy and safety of laser photocoagulation, significantly reduce the associated side effects, and shorten the duration of laser treatment.^20^ Clinical trials have also confirmed that the therapeutic efficacy of lower-energy and shorter exposure time laser photocoagulation for the treatment of DME is comparable to that of traditional laser photocoagulation.^21, 22, 23, 24^ Thus, it is reasonable to question whether thermal retinal damage is a necessary aspect of DME laser photocoagulation therapy, and how to avoid the risks and side effects associated with it.

Idealised subthreshold photocoagulation is defined as photocoagulation resulting in laser spots that are not visible under any light microscope.^25, 26^ On the basis of animal experiments and *in vitro* cell culture, several hypotheses relating to subthreshold photocoagulation DME therapy have been proposed. One is that cytokines and vasoactive factors produced by RPE have an important role in retinal vascular disease. Subthreshold photocoagulation acts on RPE and reduces the expression of cytokines and vasoactive factors, thereby decreasing macular capillary permeability.^27^ Second, subthreshold photocoagulation may change the retinal barrier. Pollack and colleagues^28^ observed the migration and proliferation of RPE cells surrounded by laser spots in pigmented rabbit after subthreshold photocoagulation. This kind of migration and proliferation promotes RPE repair. It has been suggested that subthreshold photocoagulation helps to increase the permeability of RPE in the early stage, and promotes the discharge of effusion in areas of oedema. In the later stage, RPE repair promotes metabolism and water discharge from the retina to the choroid. At the same time, subthreshold photocoagulation, which causes a relatively low level of damage to the RNFL and lower inflammatory reactions, preserves DR patients' visual function.^29^ Some clinical studies have concluded that micro-pulse diode laser subthreshold photocoagulation could improve visual acuity and alleviate DME.^30^ On the basis of the research described above, we designed a preliminary randomised clinical trial to confirm the efficiency of short-pulse threshold retinal macular grid photocoagulation for DME.

We selected binocular diffuse DME with CMT of more than 300 *μ*m as the subject of our investigation and grouped the eyes of the same patients into separate subthreshold and threshold groups, to preclude other systemic and age related diseases from confounding the results. In this clinical trial, there was a significant improvement in BCVA after 6 months of subthreshold retinal macular grid photocoagulation. Threshold photocoagulation was effective earlier than subthreshold photocoagulation, which yielded improved BCVA at 1 and 3 months. However, there was no significant difference at 6 months follow-up compared with baseline. CMT decreased in both groups, and in the subthreshold group CMT differed significantly from baseline from 3 months onwards. In patients treated with subthreshold retinal macular grid photocoagulation, both BCVA and CMT improved. With regard to DME, the therapeutic effects of subthreshold and threshold retinal macular grid photocoagulation did not differ significantly between the two groups. Possible reasons for this observation include inadequate sample size, relatively short follow-up, and lack of CMT-stratified accordance with the severity stratification in the study subjects.

Though the therapeutic effect is not as good as intravitreal anti-VEGF,^31^ laser photocoagulation remains the mainstay of therapy for DME patients. Reducing the side effects and improving the efficacy and safety of the therapy are some of the central aims of contemporary laser photocoagulation research. Our preliminary study suggests that subthreshold photocoagulation can enhance visual acuity, and alleviate macular oedema. Invisible laser spots after therapy means subthreshold photocoagulation causes a relatively low level of damage to the RNFL and lower inflammatory reactions, which preserves DR patients' visual function. Our clinical trial has limitations, including a relatively small sample size and a follow-up period of less than a year, lack of macular visual function examination such as contrast sensitivity and micro-perimetry. Prospective, large sample, multicentre, randomised, controlled studies will provide further valuable insight into the effectiveness and reliability of subthreshold photocoagulation.


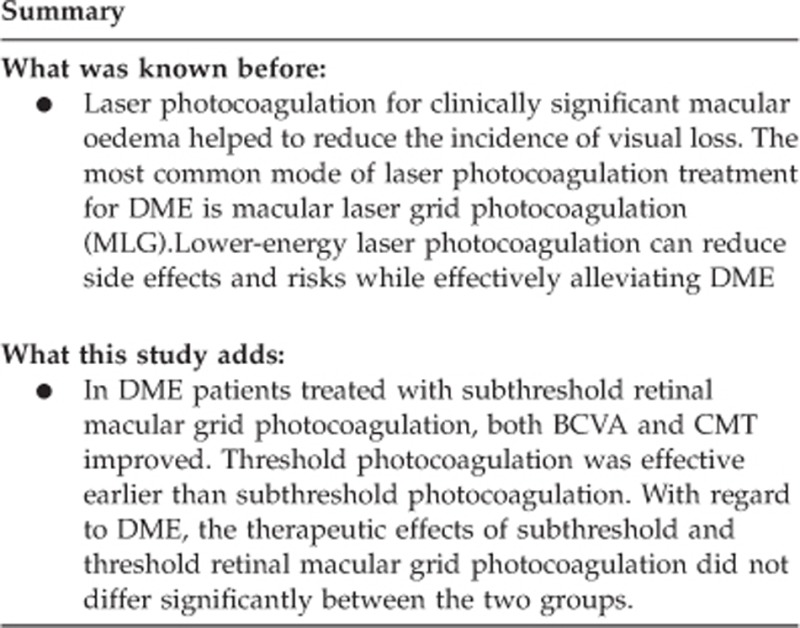


## Figures and Tables

**Table 1:** Patient characteristics for ZOC subthreshold pascal study

*Study patient*	*Age (years)*	*Sex*	*HbA1C (entry/final)*		*Average GLU (entry/final)*		*Length of DM (years)*	*Follow-up (months)*
1	67	M	6.0	6.1	6.9	4.7	2	6
2	69	M	9.5	8.2	8.2	7.2	15	6
3	58	M	7.5	7.2	9.3	8.4	8	6
4	59	F	6.8	5.9	6.7	5.3	1	6
5	56	F	8.2	8.4	7	7.5	11	6
6	65	F	5.6	5.9	6.1	7.0	11	6
7	69	M	4.0	4.2	6.1	6.5	13	6
8	65	F	6.5	6.3	7.7	7.0	4	6
9	71	F	10.3	8.2	6.6	7	23	6
10	65	F	10.6	10.4	7.3	6.8	38	3
11	62	M	7.0	7.1	5.5	6.0	16	3
12	64	F	7.0	6.4	6.4	5.7	2	6
13	49	M	10.4	8.7	8.8	7.2	7	6
14	47	M	8.3	8.4	10.5	7.3	8	6
15	72	F	9.4	10.5	5.0	8	9	6
16	58	M	5.6	5.8	6.0	6.3	3	6
17	68	F	5.9	5.6	4.9	6	1	6
18	73	M	10.5	9.6	10	8.6	11	6
19	61	F	7.8	7.9	7.2	7.0	6	6
20	50	M	9.6	9.8	7.5	7.8	15	6
21	63	M	7.2	7.5	5.5	6.0	1	6
22	49	F	10.2	10.8	8.6	11	14	6
23	57	F	9.6	10.2	7.8	7.5	16	6

Abbreviations: DM, diabetes mellitus; GLU, serum glucose; HbA1C, glycosylated haemoglobin.

**Table 2:** Central macular thickness and BCVA

*Visit*	*CMT*		*BCVA*	
	*1*	*2*	*1*	*2*
Baseline	364 (291–787)	438 (272–716)	60 (18–83)	51 (15–85)
7 Days	358.5 (238–757)	404.5 (268–668)	55 (23–80)	51 (19–80)
1 Month	356 (278–792)	409 (274–699)	58 (17–80)	55 (17–79)
3 Months	340 (212–668)	415 (270–736)	59 (17–82)	57 (20–79)
6 Months	320 (213–663)	387 (245–692)	64 (21–83)	56 (18–84)

Abbreviations: BCVA, best-corrected visual acuity; CMT, central macular thickness.

Data are the median (min−max).

**Table 3:** BCVA improvements and central macular thickness decrease over study follow-up

	*BCVA*		*P*_*1*_		P-*value Between 2 Groups*
	*1*	*2*	*1*	*2*	
*BCVA improvements*					
7 Days	−1.61 (6.87)	0.70 (6.36)	0.242	0.413	0.271
1 Month	−1.19 (10.67)	2.40 (4.64)	0.394	0.001	0.221
3 Months	0.74 (9.20)	3.50 (6.38)	0.165	0.019	0.659
6 Months	4.03 (9.02)	2.17 (7.41)	0.035	0.065	0.428
					
*Central macular thickness decrease*					
7 Days	−3.42 (62.27)	−25.85 (43.13)	0.626	0.055	0.787
1 Month	−15.05 (51.58)	−14.80 (104.45)	0.158	0.465	0.664
3 Months	−49.67 (69.17)	−23.29 (89.89)	0.001	0.156	0.126
6 Months	−62.30 (102.52)	−23.63 (80.99)	0.005	0.040	0.399

Abbreviation: BCVA, best-corrected visual acuity.

Data are mean±SD.

*P*_*1*_ are the *P*-value of data compared with baseline.
